# Integrative circRNA landscape of intrauterine adhesions: putative ceRNA axes and circRNA-associated splicing usage linked to contractility and immunity

**DOI:** 10.3389/fmolb.2026.1763980

**Published:** 2026-05-07

**Authors:** Yingqing Chen, Jing Jin, Ying Xiang, Yanping Wang, Shuang Wu, Ni Zhan, Mengxin Xiong, Ali Deng

**Affiliations:** 1 Hubei Provincial Hospital of Traditional Chinese Medicine, Wuhan, China; 2 College of Chinese Medicine, Hubei University of Chinese Medicine, Wuhan, China; 3 Hubei Shizhen Laboratory, Wuhan, China; 4 Affiliated Hospital of Hubei University of Chinese Medicine, Wuhan, China; 5 Guangshui Traditional Chinese Medical Hospital, Suizhou, China

**Keywords:** circular RNA, ceRNA, circRNA-associated splicing, intrauterine adhesions, RNA-binding proteins

## Abstract

**Background:**

Intrauterine adhesions (IUA) are fibrotic scars that impair endometrial regeneration, and compromise fertility. Emerging evidence implicates circular RNAs (circRNAs) in fibrotic remodeling, but it remains unclear how the circRNA landscape and circRNA-associated splicing programs coordinately link uterine contractility, endometrial cell-cycle control, and immune activation in IUA.

**Methods:**

To address this question, we reanalyzed rRNA-depleted RNA sequencing data from IUA and controls (GSE224093) to assemble a high-confidence circRNA catalog using four independent circRNA callers, identify differential expression, and construct direction-consistent circRNA–miRNA–mRNA competing endogenous RNA (ceRNA) circuits. Gene set enrichment and CIBERSORT-based deconvolution were combined to relate circRNA modules to smooth muscle contractility, proliferative programs, and macrophage phenotypes. CircRNA-associated splicing (CAS) usage was quantified with SUVA to detect IUA-related shifts in back-splicing *versus* canonical splicing, and CAS–RNA-binding protein (RBP) co-expression networks were delineated using differentially expressed RBPs. Selected circRNAs, mRNAs, and RBPs were validated in an independent cohort of IUA and non-IUA endometrial samples by back-splice junction–spanning RT-qPCR and Western blotting.

**Results:**

We identified 1,724 high-confidence circRNAs arising from 1,143 host genes. ceRNA integration highlighted an upregulated circRNA, hsa-KDM4B_0007, converging on smooth muscle contractility genes MYH11 and PPP1R12B, and downregulated circRNAs hsa-LPAR3_0001 and hsa-PPFIA1_0013 converging on cell-cycle regulators CDC6 and CDCA5. Immune deconvolution indicated expansion of both M1 and M2 macrophages in IUA, and several DECs (e.g., hsa-PLOD2_0001 and hsa-FAM13B_0009) were tightly correlated with M1-enriched inflammatory signatures. CAS profiling uncovered widespread alterations in circRNA-associated splicing usage within pathways related to uterine contractility, EGFR signaling, and epithelial–mesenchymal transition. Integration with differentially expressed RBPs revealed a CAS–RBP network in which RBPs such as EXO1 and SORBS1 tracked with CAS events in EGFR adaptor genes (e.g., GAB1, PTK2) and other fibrogenesis-related regulators. Back-splice junction–spanning RT-qPCR confirmed the dysregulation of multiple DECs and their putative target mRNAs, and protein-level changes of selected RBPs were verified in clinical samples.

**Conclusion:**

This study provides a mechanistic circRNA map that links ceRNA circuitry with CAS remodeling. The ceRNA and CAS–RBP modules we delineate generate testable hypotheses on circRNA-driven fibrotic remodeling and nominate circRNAs, CAS events, and RBPs as potential biomarkers and therapeutic entry points for IUA.

## Background

Intrauterine adhesions (IUA), a common gynecological disease, frequently arise from surgical trauma to the endometrium, caused by procedures such as uterine curettage. The formation of IUA can lead to endometrial fibrosis, which can cause infertility, abnormal menstrual periods, and even abortion, seriously affecting women’s fertility and quality of life (Khan, 2023; Hooker et al., 2014; Zhao and Hu, 2024). IUA is often associated with post-injury inflammatory responses and fibrosis processes, in which pro-inflammatory macrophages, transforming growth factor-β (TGF-β), matrix metalloproteinases (MMPs), and tissue inhibitors of metalloproteinases (TIMPs) play important roles (Ang et al., 2023; Wu et al., 2022; Lv H. et al., 2023). Conventional management strategies for IUA, such as adhesiolysis and estrogen supplementation, frequently fail to restore fertility in affected patients fully. Recently, mesenchymal stem cells (MSCs), a promising candidate for stem cell-based therapy, have demonstrated significant potential in enhancing endometrial tissue repair and regenerative capacity (Liu H. et al., 2024; Ma et al., 2021). Despite progress in research, the high recurrence rate after IUA treatment still necessitates more effective prevention and treatment strategies. Additionally, the primary molecular targets and their regulatory mechanisms in the pathological process of IUA remain not fully understood. Further research is required to unveil these mechanisms and enable the development of more precise therapeutic approaches.

Circular RNAs (circRNAs) are a class of non-coding RNAs (ncRNAs) widely expressed in eukaryotes. They are formed by the back-splicing of Messenger RNAs (mRNAs) and lack a 5′cap and a 3′polyadenylate (polyA) tail (Wu et al., 2023). Although circRNAs contribute to the pathogenesis of various human diseases, the research in IUA is relatively limited. Notably, Xie *et al.* demonstrated that circPlekha7 exhibits antifibrotic properties through modulation of critical fibrosis-related markers in endometrial stromal cells (ESCs) modeling of IUA (Xie et al., 2020). In addition, research investigating human umbilical cord mesenchymal stem cells (HuMSCs) for IUA treatment revealed that circPTP4A2 on HuMSCs-SF-SIS scaffold played a pivotal role in mediating endometrial repair processes, which provided critical mechanistic insights through comprehensive circRNAs analysis (Zheng et al., 2022). Xing *et al.* reported elevated expression of Hsa_circ_0079474 in IUA tissues relative to normal controls, with functional evidence suggesting its potential role in promoting epithelial-mesenchymal transition (EMT), which provides an essential clue for further study of the role of circRNAs in IUA (Xing et al., 2024). Current research mainly focuses on the expression differences of circRNAs and their potential influence on the occurrence and development of intrauterine adhesion *via* competing endogenous RNA (ceRNA) networks (Peng et al., 2023; Chang et al., 2025), while studies on the mechanisms related to their splicing are relatively limited; moreover, existing studies are still insufficient in the systematic identification and functional investigation of IUA-associated circRNAs on a genome-wide scale. Most investigations of circRNAs in IUA to date have relied on microarray-based datasets and have mainly catalogued differentially expressed circRNAs (DECs), whereas high-throughput RNA sequencing has the potential to uncover a much broader repertoire of previously unannotated circRNAs and to enable more comprehensive, multi-layered analyses and functional inference beyond simple expression-level changes.

CircRNAs are produced by a unique RNA splicing process termed “back-splicing”, which involves the covalent joining of downstream 5′splice sites to upstream 3′splice sites. Unlike ordinary forward-splicing, this mechanism is a key step in circular RNA formation (Eg et al., 2018; Zhang et al., 2016; Li et al., 2020). A subset of circRNAs modulates gene expression post-transcriptionally, serving as molecular sponges for microRNAs (miRNAs)/RNA-binding proteins (RBPs) or competing with mRNA molecules.

To fill this gap that functions and roles of DECs in IUA, we generated an integrative circRNA atlas for IUA. We hypothesise that specific circRNA-mediated ceRNA networks and circRNA-associated alternative splicing(CAS) events synergistically regulate endometrial fibrosis, smooth muscle contraction, and the immune microenvironment in IUA. We first established a consensus set of circRNAs and their differential expression from BSJ counts. We next constructed direction-consistent, prediction-supported circRNA–miRNA–mRNA axes, and estimated immune context with CIBERSORT to pinpoint circRNAs associated with macrophage polarization. We then quantified CAS usage, identified differential events, and integrated these with differentially expressed RBPs through correlation networks. Finally, we validated prioritized targets by BSJ-spanning Reverse transcription quantitative polymerase chain reaction(RT-qPCR) and Western blotting (WB). Collectively, this framework delivers a combined view of ceRNA circuitry and splicing-usage alterations, nominating mechanistically plausible and clinically relevant candidates for IUA.

## Materials and methods

### Process and alignment of RNA-seq data

We retrieved publicly available rRNA-depleted RNA-seq datasets comprising 14 tissue specimens. This dataset included endometrial samples from 7 cases of severe IUA and seven normal controls, sourced from the Gene Expression Omnibus (GEO) database (GSE224093). Detailed clinical characteristics of this cohort have been previously described (Yao et al., 2023). The raw reads exhibited high quality, confirmed through assessment with FastQC (v0.12.1) (Simon, 2010). Prior to circRNA identification, we conducted read alignment to the human GRCh38 genome. To meet the specific computational demands of different circRNA detection algorithms, we implemented three specialized alignment tools: HISAT2 (v2.2.1) (Kim et al., 2019) (utilized for FindCirc (Memczak et al., 2013)), STAR (v2.7.1b) (Dobin et al., 2013) (used for circRNA_finder (Westholm et al., 2014) and CIRCexplorer2 (Zhang et al., 2016)), and BWA (v0.7.19) (Li and Durbin, 2009) (applied for CIRI2 (Gao et al., 2018)).

### CircRNA prediction and annotation

The recognition of circRNAs is achieved through detecting “back-spliced reads” (BSJ). Because individual circRNA identification algorithms exhibit significant variance and can produce false positives, we implemented a multi-algorithm strategy to ensure robust detection. We utilized four widely-recognized prediction tools based on distinct alignment strategies: FindCirc (Memczak et al., 2013), circRNA_finder (Westholm et al., 2014), CIRCexplorer2 (Zhang et al., 2016), and CIRI2 (Gao et al., 2018). To effectively balance sensitivity and specificity and mitigate the false-positive rates inherent to high-throughput sequencing, we established a strict consensus threshold: the final high-confidence circRNA dataset included only those predicted concordantly by two or more independent tools. Furthermore, to filter out potential sequencing noise and prioritize circRNAs with stable biological relevance, candidate circRNAs were required to be expressed in a minimum of five samples to be included in downstream analyses. We integrated information from five well-established circRNA databases, namely, circAtlas (Wu et al., 2020), circBase (Glažar et al., 2014), circRNADb (Chen et al., 2016), deepbase2 (Zheng et al., 2016), and circpedia2 (Dong et al., 2018). Predicted circRNAs were meticulously annotated based on this comprehensive database amalgamation.

### CircRNA quantification and differential analysis

Spliced reads per billion mapped reads (SRPBM) (T et al., 2017) represents the standardized expression of each circRNA, that is, the number of back-spliced reads on each circRNA in one billion reads. The formula follows: SRPBM (circRNA) = back-spliced reads (circRNA)*1,000,000,000/total number of mapped reads.

The R package TCC (v1.40.0) (Sun et al., 2013) was applied to screen out the raw BSJ reads for detected DECs. CircRNAs meeting both statistical (p ≤ 0.05) and magnitude (|log2FC| ≥ 1) thresholds were classified as differentially expressed.

### Identification of differentially expressed genes (DEG)

For the detection of DEG, we employed DESeq2 for statistical analysis of unprocessed count data (Love et al., 2014). The fold change (|log_2_FC| ≥ 1) and FDR ≤ 0.05 were set as the dual criteria for identifying genes with significant expression changes. Using a catalog of 2,141 RBPs derived from four previous reports, we selectively filtered the expression profiles of DERBPs from all DEGs (Castello et al., 2012; Castello et al., 2016; Gerstberger et al., 2014; Hentze et al., 2018).

### CeRNA network construction of circRNA, microRNA, and DEG

Given the known role of circRNAs as miRNA sponges, we employed two methods to predict target relationships between miRNAs and DECs. The first method involved Miranda (https://anaconda.org/bioconda/miranda) computationally identifying potential miRNA-circRNA interactions, with only high-confidence pairings (Miranda score≥160) being retained for further analysis. The second method utilized Rnahybrid (https://bibiserv.cebitec.uni-bielefeld.de/rnahybrid/) to retain miRNA-circRNA pairs with a p-value ≤0.01. The final miRNA-circRNA target relationships were determined by taking the overlapping results from these two methods. Subsequently, we integrated miRDB (http://mirdb.org) and TargetScan (http://www.targetscan.org) to identify microRNA targets. Ultimately, we constructed the ceRNA network comprising DE circRNA-miRNA-mRNA using Cytoscape software (v3.10.0, https://cytoscape.org/).

### Cell-type quantification and Co-expression analysis

We applied the CIBERSORT algorithm (Newman et al., 2015) (v1.03) with the default parameter to estimate immune cell fractions based on FPKM values of each expressed gene. Twenty-two human immunological cell phenotypes were analyzed in this research, including seven T lymphocyte subsets [CD8 T cells, naïve CD4 T cells, memory CD4 resting T cells, memory CD4 activated T cells, follicular helper T cells, regulatory T cells (Tregs), and T cells gamma delta]; naïve and memory B cells; plasma cells; resting and activated NK cells; monocytes; macrophages M0, M1, and M2; resting and activated dendritic cells; resting and activated mast cells; eosinophils; and neutrophils. A co-expression network was constructed to analyze relationships between immune cell proportions and DECs, retaining only significant correlations (|Pearson’s correlation| ≥ 0.6 and p-value ≤0.05).

### Identification of alternative splice sites and the usage of circular RNA

The SUVA pipeline was employed to quantify differential circRNA splicing events in IUA *versus* normal samples, as previously described (Cheng et al., 2021). Briefly, this pipeline systematically evaluates the variation in the selection of competing splice sites for each circRNA to identify circRNA-associated splicing (CAS) events. Based on the resulting RNA products, these events are further categorized into circRNA-circRNA (circ-circ) or circRNA-linear (circ-linear) alternative splicing events. To identify functionally relevant CAS events, we calculated the splicing ratio (Percent Spliced In, PSI) for each identified site. Significant differential splicing events between the IUA and control groups were strictly defined using dual thresholds: a statistical significance of p-value <0.05 and an absolute minimum difference in splicing ratio (|ΔPSI|) > 0.15.

### Co-disturbed analysis of RBP and CAS

We created the co-disturbed network by investigating the connection between IUA-related DERBPs and the splicing ratios of CAS. We applied Pearson’s correlation coefficient analysis to establish relationships within this network. Only correlations with an absolute value of Pearson’s correlation coefficient ≥0.7 and a *p-value* ≤0.01 were retained for further analysis and interpretation.

### Functional enrichment analysis and other analyses

To identify functional categories of genes, we employed the clusterProfiler package (v4.6.2) (Wu et al., 2021), which enabled us to determine Gene Ontology (GO) terms and Kyoto Encyclopedia of Genes and Genomes (KEGG) pathways. We performed principal component analysis (PCA) with the R package factoextra (https://cloud.r-project.org/package=factoextra) to visualize sample clustering patterns based on the first two components. Euclidean distance-based clustering was implemented using the pheatmap package (https://cran.r-project.org/web/packages/pheatmap/index.html) in R. After normalizing gene expression values using Tags Per Million (TPM) across all samples, we utilized the in-house sogen tool to graphically represent alternative splicing patterns in next-generation sequencing data alongside genomic annotations.

### Tissue samples

Six human IUA tissue and six human non-IUA tissue samples were collected from patients undergoing transvaginal hysteroscopic surgery in Hubei Provincial Hospital of Traditional Chinese Medicine. Patients diagnosed with IUA by hysteroscopy and an American Fertility Society (AFS) score ≥3 were selected for IUA tissue samples (Li et al., 2025a). Patients undergoing hysteroscopy for infertility, endometrial polyps, or similar conditions, but whose postoperative pathological examination was normal and showed no uterine cavity adhesions, were selected as non-IUA tissue samples. Detailed information of clinical patients is shown in Table 1. The Ethics Committee at Hubei Provincial Hospital of Traditional Chinese Medicine reviewed and approved all study protocols (Ethics ID: HBZY2025-C66-01). The study protocol adhered strictly to the Declaration of Helsinki’s ethical standards. No participants received hormone therapy during the study period. Endometrial biopsies were collected during the early proliferative phase (cycle days 6–10) for all participants. IUA tissue was taken from the adhesion zone area in the uterus, and normal endometrium was taken from the non-adherent and pathologically normal area of the uterus. Following collection, all tissue samples were immediately stored at −80 °C for subsequent analysis.

**TABLE 1:** Baseline demographic and clinical characteristics of patients in the two study groups.

Index	IUA group	Normal group	*P*
Age(yesrs)	29.67 ± 5.32	34.00 ± 4.24	0.1497
BMI(kg/m^2^)			
Underweight	0	0	
Normal	3/6(50%)	2/6(33.3%)	
Overweight	3/6(50%)	4/6(66.7%)	
Obese	0	0	
Pregnancy times (times)	2(1–4)	1(0–3)	0.1693
Number of abortions (times)	1(1–3)	0(0–2)	0.0653
AFS score	5.67 ± 1.37	0.00 ± 0.00	<0.0001

Data are presented as median, or as mean ± standard deviation (percentage). BMI, body mass index; BMI: underweight (BMI < 18.5), normal (18.5 ≤ BMI < 25), overweight (25 ≤ BMI < 30) and obese (BMI ≥ 30).

### RNA extraction and RT-qPCR validation

One mg of endometrial tissue was taken from each of the two groups. RNA isolation was performed from these samples using Trizol reagent (Ambion, 15596026). To synthesize complementary DNA (cDNA), reverse transcription of RNA was performed using the Hifair® III 1st Strand cDNA Synthesis SuperMix (YEASEN, China), and the resultant cDNA was preserved for subsequent applications. Amplification of cDNA was conducted using the SuperMix for qPCR (gDNA digester plus) and Hieff® qPCR SYBR® Green Master Mix (both obtained from YEASEN, China). The qPCR primers for circRNA span the back-splice junction (BSJ), and the primer sequences are shown in Table 2. Statistical analysis was performed using 2^−ΔΔCt^. Each group tests six samples repeatedly, with each sample being tested three times.

**TABLE 2:** Primer sequences of selected circRNAs, DEGs, and human GAPDH for RT-qPCR.

circRNA and DEG	Sense primer	Antisense primer
hsa-FAM13B_0009	GATGGTGTTAATCTGTCCTCTGAGT	CATGGTCTAGAGAAGAGCCAAAGC
hsa-KDM4B_0007	CACATTTCCCTACGGCTACCAC	ATGTTCCACTGGGCCACGTC
hsa-PPFIA1_0013	TGAAGCTGAGGCTGGCCAT	CTGTGGAGTTCCTTATGTCCCTGAT
hsa-LPAR3_0001	TGTCCAACCTCATGGCCTTC	ATTGTGGAGAAGTGAACATCCTAAG
hsa-PLOD2_0001	AGAGTGGTTACGGTCCTTGGTC	TGACAGATCCTTCTTTCTTCACAGT
MYH11	AGCGGCAACTCGTGTCCAA	CCATTTCGGCTTTGAGCATT
PPP1R12B	CTTCTGGCAAGAGGTGCTGATA	GGTTTACATTGGCTCTGTTCTCC
CDC6	ACAAATGTCCAAACCGTAACCTG	GTCAAATACCAATCTTCGTCCCT
CDCA5	CCGAGCATCCTCCCTGAAAT	CAAGAAAAAGGAAATCCTAGGGC
EXO1	ATTGCCTCGTGGCTCCCTAT	CCAAGCTGTCTGCACATTCCT
SORBS1	ACTGCCAGACCTCCAACACCT	ATCGGTACTCGAAACAGCTCCC
GAPDH	GGAAGCTTGTCATCAATGGAAATC	TGATGACCCTTTTGGCTCCC

### Western blot

Cut the endometrial tissue into tiny pieces. Add 10 μL of lysate per 1 mg of tissue and use a tissue homogeniser to fully grind to extract the total protein. The protein concentration was determined using the BCA method, yielding results of 1–5 μg/μL. After centrifugation to obtain the target protein, heat denaturation was performed, followed by aliquoting and freezing. Samples must be thawed and thoroughly mixed before loading. Prepare the gel for the kit using 10% PAGE gel without staining. The voltage used during electrophoresis is constant at 300V. Electrophoresis was performed using 20 µg of sample, and the 400 mA constant flow membrane was transferred for 15–30 min after the electrophoresis (Li et al., 2025b). Block with an appropriate amount of low background protein-free rapid blocking solution in a clean incubator. After 1×PBST washing, add primary antibody (MYH11, 21404-1-AP, Proteintech, 1:1000; PPP1R12B, 13366-1-AP, Proteintech, 1:5000; CDC6, 11640-1-AP, Proteintech, 1:500; CDCA5, 67418-1-Ig, Proteintech, 1:1000; Ki67, R381101, Proteintech, 1:800; CD68, 25747-1-AP, Proteintech, 1:4000; CD86, A21198, Proteintech, 1:2000; CD163, 68218-1-Ig, Proteintech, 1:20000; KLHL24, 680361, Proteintech, 1:500; EXO1, 16253-1-AP, Proteintech, 1:2000; SORBS1, 13854-1-AP, Proteintech, 1:500; β-Actin, 66009-1-Ig, Proteintech, 1:10000) incubated overnight at 4 °C. After washing the membrane with 1× PBST 3 times, add the secondary antibody (Goat anti-Rabbit IgG,BL003A,Biosharp; Goat anti-mouse IgG,SAB43714,bioswamp,1:20000) and incubate at room temperature for 1 h. The ECL luminescent solution was applied to the front of the film, and it was fully in contact with it. It was exposed in a dark room, and the image was acquired. group tests three samples repeatedly, with each sample being tested three times. Each group tests three samples repeatedly, with each sample being tested one times.

### Statistical analysis

The experimental statistical analyses were performed using SPSS version 30.0, with continuous variables presented as means ± standard deviations(SDs). Statistical differences between groups were assessed *via* the independent-sample t-test, considering p-value <0.05 as statistically significant.

## Results

### CircRNA expression dynamics in IUA pathogenesis

**FIGURE 1:**
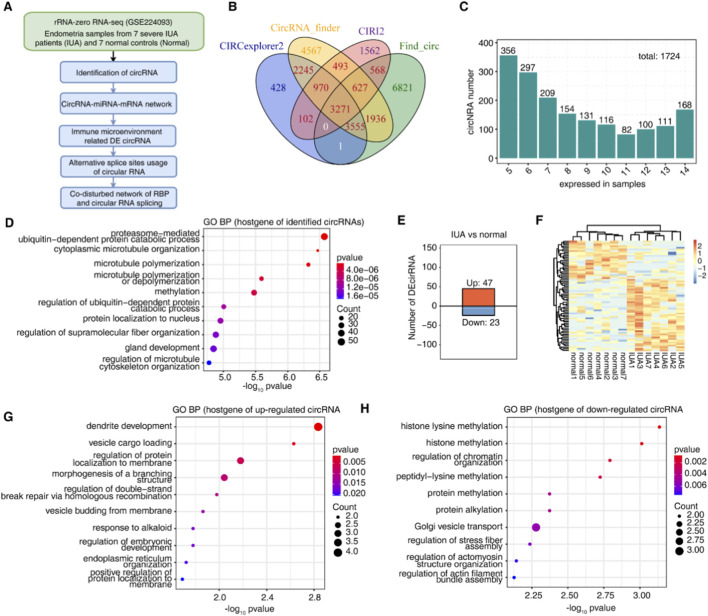
**(A)** Diagram outlining the experimental approach and analytical workflow. **(B)** Four established circRNA detection tools (FindCirc, circRNA_finder, CIRCexplorer2, and CIRI2) were employed for circRNA identification. Next, for downstream analysis, circRNA was detected concordantly by two or more tools as reliable candidates for downstream processing. **(C)** The bar graph depicts the circRNAs expressed concurrently across different sample quantities. CircRNAs detected in at least five samples were used for further study. **(D)** Dot plot illustrating the highly enriched GO biological processes associated with host genes encoding all circRNAs. **(E)** Bar plot showing the 45 upregulated circRNAs and 24 downregulated circRNAs comparing IUA with normal samples. **(F)** DECs are displayed according to their expression patterns in the heatmap diagram. **(G)** The Dot plot displays significantly enriched GO biological processes among parental genes of IUA-associated upregulated circRNAs. **(H)** Dot plot presenting the most enriched GO biological process among parental genes of IUA-associated downregulated circRNAs.

To systematically identify and investigate circRNAs in IUA, we obtained published RNA sequencing data from the rRNA deletion library of endometrial samples from the GEO database (GSE224093), including seven endometrial tissue samples with severe IUA and seven normal endometrial tissue samples as controls (Figure 1A). Currently, there are many analytical pipelines to predict circRNAs based on deep sequencing datasets. The most commonly used ones include find_circ, circRNA_finder, CIRCexplorer2, CIRI, map splice, *etc.* However, significant differences were observed among the different circRNA identification algorithms, with any combination of two algorithms significantly reducing the false positive rate and improving the result accuracy. Therefore, it is recommended that multiple algorithms be combined to achieve reliable prediction (Ebbesen et al., 2016). Four methods were used to identify circRNAs in IUA, among which circRNAs identified by two methods were considered credible circRNAs, and a total of 13,768 circRNAs were obtained (Figure 1B). Furthermore, we selected 1,724 circRNAs co-expressed in at least five samples for downstream analysis (Figure 1C; Supplementary Table S1). Next, we analyzed the basic characteristics of these circRNAs (Supplementary Figure S1A-E). Interestingly, most circRNAs were detected in the IUA and control groups (1,706/1,724) (Supplementary Figure S1F).

Furthermore, we wanted to understand the function of the genes of the circRNAs identified in IUA and normal, and the gene ontology biological process (GO-BP) pathway enrichment was conducted on the host genes of the identified circRNAs (Figure 1D). Our analysis demonstrated that the majority of these genes participate in proteasome-mediated ubiquitin-dependent protein catabolism, cytoplasmic microtubule organization, microtubule polymerization or depolymerization, gonadal development, regulation of supramolecular fibrous organization, and regulation of ubiquitin-dependent protein catabolism. Subsequently, we analyzed the DECs in the two groups of samples (Figures 1E,F; Supplementary Table S2). The results showed differential expression of 70 circRNAs in IUA patients relative to controls, with 47 exhibiting upregulation and 23 showing downregulation, implying their potential involvement in IUA pathogenesis. Functional enrichment analysis revealed distinct pathway associations: host genes of upregulated circRNAs showed significant involvement in dendritic morphogenesis and vesicular trafficking (Figure 1G), while those of downregulated circRNAs were predominantly linked to histone methylation processes (Figure 1H).

### The IUA-associated circRNA-miRNA-mRNA network reveals the potential involvement of circRNA in the contraction and regenerative function of the endometrium

**FIGURE 2:**
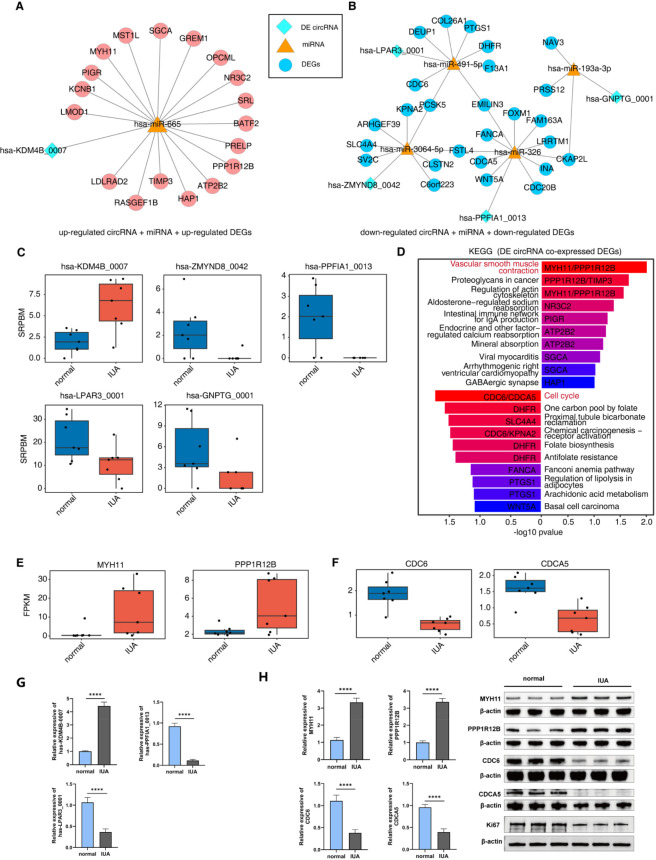
**(A and B)** The ceRNA network was established using DECs, miRNAs, and DEGs. Predictions of miRNA-circRNA target relationships were made using both Miranda and Rnahybrid. Additionally, Potential miRNA-mRNA interactions were predicted by integrating data from the miRDB (http://mirdb.org) and TargetScan (http://www.targetscan.org). This analysis identified regulatory relationships between miRNAs and DEGs. Upregulated circRNAs and upregulated DEGs are presented in Panel A, while downregulated circRNAs and downregulated DEGs are shown in Panel B. **(C)** Boxplot showing the expression level (SRPBM) of circRNA five DECs involved in Fig. A–B. **(D)** Bar graphs showing the top enriched KEGG pathways among upregulated and downregulated DEGs of circRNA-miRNA-mRNA network in figure A–B. **(E)** Boxplot displaying expression patterns (FPKM) of MYH11 and PPP1R12B. **(F)** Boxplot displaying expression patterns (FPKM) of CDC6 and CDCA5. **(G)** RT-qPCR shows a significant expression level of 3 DECs (hsa-KDM4B_0007, hsa-PPFIA1_0013, hsa-LPAR3_0001). *p < 0.05,**p < 0.01,***p < 0.001 and ****p < 0.0001. **(H)** RT-qPCR and WB confirming the differential expression of four DEGs (MYH11, PPP1R12B, CDC6, and CDCA5), and WB detecting the expression of Ki67 protein.

In recent years, ncRNAs, particularly circRNAs and miRNAs, have been widely investigated in studies (Toden et al., 2021). Functioning as miRNA sponges, circRNAs can competitively interact with miRNA response elements, effectively blocking the repression of miRNA-regulated genes. Based on this, we hypothesized that circRNAs associated with IUA could regulate mRNA expression through miRNAs. Elevated circRNA levels enhance their miRNA sequestration capacity, thereby attenuating miRNA-mediated repression of target mRNAs and promoting their expression. Similarly, the decreased expression of circRNAs minimizes the number of miRNAs absorbed by it, which will aggravate the inhibition of miRNAs on the target mRNAs, ultimately reducing the expression levels of the target mRNAs. To verify our speculation, we constructed the ceRNA regulatory network of DEcircRNA-miRNA-mRNA (Figures 2A,B; Supplementary Table S3), in which we identified a total of 851 differentially expressed mRNAs (DEGs), with 542 upregulated genes and 309 downregulated genes (Supplementary Figure S2A). Upregulated DEGs were predominantly associated with muscle cell differentiation and muscle contraction function (Supplementary Figure S2B), whereas the downregulated DEGs showed significant enrichment in biological processes involved in cell replication and division processes (Supplementary Figure S2C). The increased circRNA hsa-KDM4B_0007 in IUA may increase the expression of numerous mRNA through hsa-miR-665. Similarly, the circRNAs hsa-ZMYND8_0042, hsa-PPFIA1_0013, hsa-LPAR3_0001, and hsa-GNPTG_0001, whose expression was decreased in IUA, led to the reduced expression of numerous mRNAs, possibly through hsa-miR-3064-5p, hsa-miR-326, hsa-miR-491-5p, and hsa-miR-193a-3p, respectively (Figures 2A–C).

Subsequently, GO-BP analysis was performed on upregulated transcripts to elucidate the biological roles of these differentially expressed genes. Significant enrichment was observed in processes including muscle contraction, positive regulation of atypical NF-kappaB signal transduction, myofibril assembly, striated muscle cell development, striated muscle cell differentiation, ERBB signaling pathway, muscle cell differentiation, and so on. Pathway analysis identified significant enrichment of downregulated mRNAs in biological processes related to synaptic signal modulation, inhibitory synapse formation, stimulated fibroblast proliferation, cell junction organization, and neurodevelopmental regulation (Supplementary Figure S2D). KEGG showed that the most enriched pathway of upregulated mRNAs potentially regulated by circRNAs was the smooth muscle contraction pathway, and the most enriched pathway of downregulated mRNAs was the cell cycle pathway (Figure 2D). The above ceRNA network analysis and functional enrichment analysis have initially revealed that IUA-associated circRNAs may regulate endometrium-related biological processes (e.g., endometrial contraction and regeneration) by targeting specific mRNAs. Notably, IUA is pathologically characterized by endometrial damage, fibrosis, and adhesion formation—a process closely intertwined with both endometrial dysfunction and abnormal uterine smooth muscle behavior. As two distinct but functionally interdependent tissues of the uterus, the endometrium and uterine smooth muscle may form a “co-regulatory loop” in IUA progression (Lv H. et al., 2023). Given this pathological correlation between the two tissues in IUA, together with the uterus’ structural feature of being mainly composed of smooth muscle and the key demand for restoring endometrial function in IUA treatment, we further conducted in-depth analysis and experimental verification on core pathways and target genes (including those involved in smooth muscle function and endometrial regeneration). We found that MYH11 and PPP1R12B, genes related to smooth muscle contraction, may be potential regulatory targets of circRNAs hsa-KDM4B_0007 (Figure 2E). The potential inhibitory function of downregulation of circRNAs on cell cycle genes CDC6 and CDCA5 may lead to impaired endometrial regeneration (Figure 2F). To verify the above results, we randomly selected three significantly DECs (hsa-KDM4B_0007, hsa-PPFIA1_0013, hsa-LPAR3_0001) for RT-qPCR verification (Figure 2G), and four DEGs (MYH11, PPP1R12B, CDC6 and CDCA5) for RT-qPCR and WB verification(Figure 2H). Notably, the results showed that compared with the normal group, the mRNA expression levels of genes CDC6 and CDCA5 were significantly downregulated in the IUA group (CDC6: FC = 0.3314, log2FC = −1.5933, FDR<0.001; CDCA5: FC = 0.3981, log2FC = −1.3289, FDR<0.001), suggesting that the downregulation of these two genes may be related to the occurrence and development of the disease. To assess the overall proliferative activity of the organisation, we further examined the levels of Ki67 protein, and WB results indicated that Ki67 protein expression was significantly decreased in the IUA group.

### Immune cell infiltration and its correlation with DECs in IUA

**FIGURE 3:**
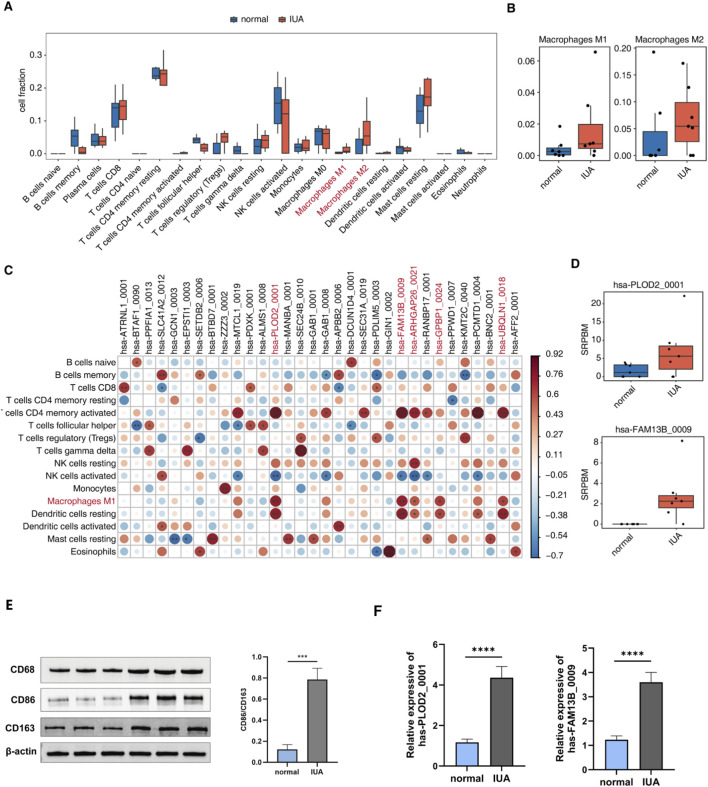
Immune cell infiltration and its correlation with DECs in IUA. **(A)** Boxplot showing immune cell type proportions in normal *versus* IUA endometrial samples using CIBERSORT. **(B)** Box plot showing the cell fraction of M1 and M2 macrophages in normal *versus* IUA endometrial samples. **(C)** The dot plot illustrated associations among immune cell fraction and DECs. Different colors show the associations of immunocyte-circRNA, and significant ones were marked by stars. *p < 0.05,**p < 0.01,***p < 0.001 and ****p < 0.0001. **(D)** Boxplot displaying expression level (SRPBM) of hsa-PLOD2_0001 and hsa-FAM13B_0009. *p < 0.05,**p < 0.01,***p < 0.001 and ****p < 0.0001. **(E)** WB detecting the expression of protein (CD68, CD86, CD163) and calculating the CD86/CD163 greyscale ratio. **(F)** RT-qPCR shows a significant expression level of DECs (hsa-PLOD2_0001, hsa-FAM13B_0009). *p < 0.05,**p < 0.01,***p < 0.001 and ****p < 0.0001.

Evidence has shown that immune cells, especially macrophages, are involved in the profibrotic process of intrauterine adhesion. Therefore, we also analyzed this project’s immune cell types in endometrial tissues. Initial immune profiling using CIBERSORT revealed significant alterations in leukocyte composition between IUA and control endometrial tissues, with multiple immune cell populations demonstrating differential abundance patterns (Figure 3A). The proportion of M1 and M2 macrophages, which were of great interest to us, was increased in patients with IUA, consistent with previous reports (Figure 3B). Furthermore, Our investigation of DECs–immune cell relationships identified five circular RNAs showing strong positive correlations with M1 macrophage abundance. The five circRNAs were hsa-PLOD2_0001, hsa-FAM13B_0009, hsa-ARHGAP26_0021, hsa-GPBP1_0024, hsa-UBQLN1_0018 (Figures 3C,D; Supplementary Figure S3A). We also predicted the potential target miRNA of these circRNAs (Supplementary Figure S3B). To verify the above results, we used WB to detect CD68 (macrophages), CD86 (M1), and CD163 (M2), which was consistent with our predicted results of the M1 and M2 cell ratios (Figure 3E). Meanwhile, we quantified the macrophage polarization balance by calculating the CD86/CD163 greyscale ratio (M1/M2 polarization index) and found that this ratio was significantly increased in the IUA group (Figure 3E). Subsequently, we selected two significantly DECs (hsa-PLOD2_0001, hsa-FAM13B_0009) for RT-qPCR verification (Figure 3F). Hsa-PLOD2_0001 and hsa-FAM13B_0009 were upregulated to a statistically significant degree, which was consistent with our analysis.

### Identification of highly conserved alternative splice sites and usage of circRNA in IUA

**FIGURE 4:**
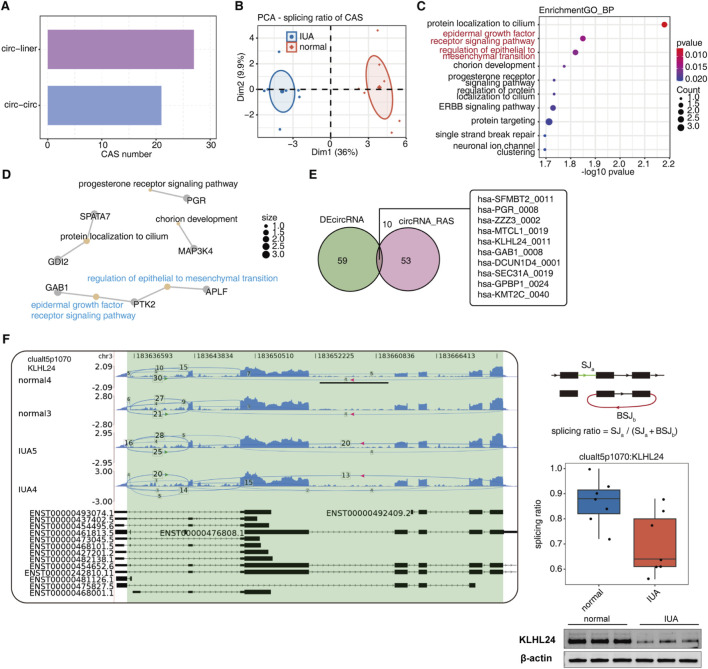
**(A)** Bar plot showing some regulated alternative splice site usage events of circular RNA detected by SUVA comparing IUA with normal samples. **(B)** PCA of splicing ratios for CAS events. Each group is depicted with a confidence ellipse. **(C)** A dot plot illustrating the highly enriched GO biological processes associated with host genes of all CAS. **(D)** The network illustrates the five most enriched GO biological processes linked to the host genes encoding circRNAs with CAS. **(E)** The Venn diagram displays the intersection of DECs between IUA and normal samples or those that contain CAS. **(F)** The reads distribution chart shows the CAS of clualt5p1070:KLHL24. The model diagram and splicing ratio profile shown by the boxplot, along with the WB validation of KLHL24, can be seen in the right panel.

For systematic mapping of the alternative regulation of circRNAs linear splice sites and back splicing, we employed SUVA, a recently developed splicing analysis platform, to detect CAS sites between IUA patients and normal controls. According to the RNA products produced using splice sites, splicing events were divided into circ-linear and circ-circ two types (Supplementary Figure S4A), of which 27 circ-linear events and 21 circ-circ events were detected (Figure 4A; Supplementary Figure S4B-C; Supplementary Table S4). PCA clustering using splicing ratios from these 48 CAS events effectively distinguished between sample groups, demonstrating a strong association between circRNA splicing patterns and IUA pathogenesis (Figure 4B). Subsequently, GO-BP revealed that genes associated with differential splicing events were predominantly involved in key biological processes, including protein localization to cilia, epidermal growth factor receptor (EGFR) signaling pathway, regulation of epithelial to mesenchymal transition, chorion development, progesterone receptor signaling pathway, targeted protein delivery, ERBB signaling pathway (Figure 4C). The genes GAB1, PTK2, and APLF are involved in both EGFR signaling and epithelial-mesenchymal transition pathways, suggesting a potential role in IUA pathogenesis (Figure 4D; Supplementary Figure S4D).

Competition between back-splicing and canonical linear splicing generates both circRNA and linear RNA counterparts, with splicing efficiency variations potentially causing differential RNA expression. In this study, we interposed the DECs and the differential CAS between the IUA and normal groups and obtained 10 circRNAs (Figure 4E), which were worthy of attention. Furthermore, we used the reads distribution map to demonstrate clualt5p1070:KLHL24 (Figure 4F), which showed that clualt5p1070:KLHL24 reduced the selection of back-splice sites in the IUA group. Existing studies have shown that the E3 ubiquitin ligase KLHL24 can inhibit myofibroblast activation by mediating the degradation of vimentin, thereby reducing skin fibrosis induced by bleomycin (Liu Y. et al., 2023). These results suggest that these alternatively spliced circRNAs may play an essential role in intrauterine adhesion. Therefore, we used WB to detect the gene expression of KLHL24, verifying our predictions.

### RBP-mediated regulation of circRNA splicing in IUA

**FIGURE 5:**
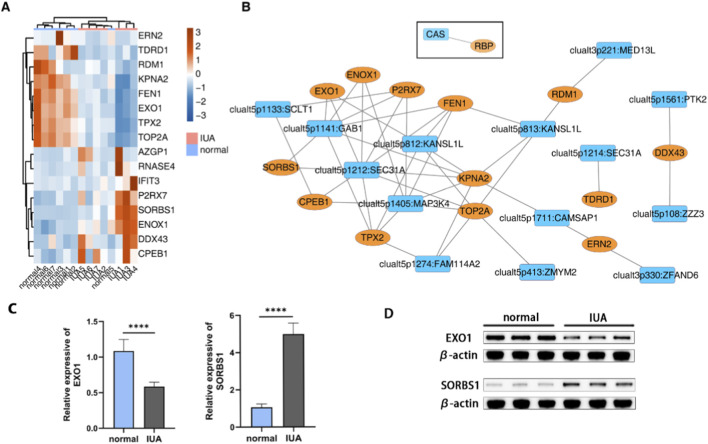
**(A)** Heatmap displaying expression patterns of differentially expressed RBPs comparing IUA with normal samples. **(B)** Co-expression analysis of DERBPs and CAS. Cutoffs of p-value ≤0.01 and Pearson coefficient ≥ 0.7 or ≤ −0.7 were applied to identify the co-expression pairs. **(C)** RT-qPCR showing expression level of EXO1 and SORBS1, *p < 0.05,**p < 0.01,***p < 0.001 and ****p < 0.0001. **(D)** WB is showing the expression levels of EXO1 and SORBS1.

Disruption of the normal function of RBPs may cause cell dysfunction by affecting the post-transcriptional regulation of RNA, including alternative splicing mechanisms. To investigate the regulatory role of IUA-related RBP genes on circRNA splice site selection, we first analyzed and identified the differentially expressed RBPs (DERBPs) in the two groups of samples, and a total of 16 DERBPs were obtained for display (Figure 5A). To analyze further the regulatory role of RBPs in circRNA splice site selection, we performed co-expression analysis of DERBPs and CAS events to predict the potential regulation of RBPs on CAS (Figure 5B). Two CAS events in the EGFR signaling pathway were clualt5p1141:GAB1 and clualt5p1561:PTK2, whose potential upstream RBPs, respectively, were EXO1, SORBS1, ENOX1, P2RX7, KPNA2, TPX2, and DDX43. Two significantly differentially expressed EXO1 and SORBS1 were randomly selected for RT-qPCR and WB validation (Figures 5C,D). Our results demonstrate that while EXO1 expression is markedly suppressed in IUA endometrium, SORBS1 shows significant elevation.

## Discussion

To our knowledge, this is the first study to use high-throughput sequencing for identifying circRNAs in patients with IUA. We used four identification methods to systematically predict circRNAs in the two groups of samples, which increased the reliability of the detection of circRNAs. However, it is worth noting that only 1,724 out of the 13,768 candidate circRNAs were expressed in at least five samples, suggesting that the expression of circRNAs has high sample heterogeneity. GO-BP analysis revealed that the 1,143 host genes associated with 1,724 circRNAs were predominantly enriched in protein degradation, microtubule polymerization, or depolymerization. These findings are similar to previous reports, alleviating endometrial fibrosis through protein degradation (Li et al., 2023). It indicates that circRNAs play a predominant regulatory role in IUA fibrogenesis.

In both IUA and normal samples, we constructed a network in which DECs potentially regulate mRNA through miRNAs. It contains five DECs, which are hsa-KDM4B_0007, hsa-ZMYND8_0042, hsa-PPFIA1_0013, hsa-LPAR3_0001, and hsa-GNPTG_0001. KEGG pathway analysis revealed that the most enriched pathway for upregulated mRNA potentially regulated by DEC was the smooth muscle contraction pathway. In contrast, the cell cycle pathway was the most enriched for downregulated mRNA. We predicted that the upregulation of hsa-KDM4B_0007 in IUA promoted the expression of smooth muscle cell contraction-related gene MYH11/PPP1R12B by adsorbing miRNA hsa-miR-665. Downregulation of hsa-LPAR3_0001 and hsa-PPFIA1_0013 in IUA may inhibit the expression of cell cycle genes CDC6/CDCA5 through miRNA hsa-miR-491-5p and hsa-miR-326. Notably, MYH11 has been strongly implicated in fibrotic tissue remodeling processes (Li et al., 2023; Ray et al., 2020). Research indicates that MYH11 is highly expressed in GFPbright cells located at the junction of the endometrium and myometrium, suggesting that it may be an important factor affecting endometrial fibrosis when the endometrium is damaged (Sohler et al., 2013) PPP1R12B modulates myosin phosphatase function, and its deficiency inhibits cardiac fibrosis development in hypertension models involving mineralocorticoid receptor activation (Ye et al., 2024). CDC6 is a genetic marker of cell proliferation that potentially regulates the cell cycle and initiates DNA replication. Its expression dominates in the endometrium during the proliferative phase (Sohler et al., 2013). These results suggest that overexpression of CDC6 may lead to cell proliferation and fibrosis. CDCA5 plays a crucial role in mitotic sister chromatid segregation and condensation, and its overexpression is frequently observed in various tumors (Zhang Y. et al., 2024). Previous studies have also shown that downregulated CDCA5 promotes smooth muscle proliferation, which promotes the process of bladder fibrosis (Di et al., 2023). Our experiments verified the increased expression of hsa-KDM4B_0007, MYH11, and PPP1R12B in endometrial tissue from IUA patients, while the expression of hsa-LPAR3_0001, hsa-PPFIA1_0013, CDC6, and CDCA5 decreased. To evaluate the overall proliferative activity of the organisation, we further assessed the levels of Ki67 protein, which is a key marker reflecting cellular proliferative activity. GO pathway analysis showed that the upregulated mRNA potentially regulated by DEC was enriched in pathways such as muscle contraction, positive regulation of non-canonical NF-kappaB signal transduction, myofibril assembly, striated muscle cell development, striated muscle cell differentiation, ERBB signaling pathway, and muscle cell differentiation. At the same time, we found that the downregulated mRNA was mainly enriched in regulating trans-synaptic signaling, inhibitory synapse assembly, positive regulation of fibroblast proliferation, cell junction assembly, and nervous system development. We observed that the genes related to the positive regulation of atypical NF-kappaB signaling were elevated in the IUA group, consistent with previous research findings. Wang’s investigation revealed upregulated inflammatory mediator NF-κB in IUA patient endometrium, with IUA animal models confirming its pathogenic contribution to adhesion formation (Wang et al., 2017). Researchers have found that electroacupuncture treatment significantly downregulated NF-κB signaling and attenuated inflammatory mediator secretion in rodent IUA models (Liu J. et al., 2023). Downregulation of hsa-miR-326 is observed in the endometrium of women with IUA. Mechanistically, miR-326 exerts antifibrotic effects through targeted suppression of TGF-β1/Smad3-mediated signaling (Ning et al., 2018).

Subsequently, CIBERSORT-based immune profiling revealed elevated proportions of both M1 and M2 macrophage subsets in IUA patients compared to healthy controls, which was also confirmed by WB detection. Consistent with previous research findings, CD301 macrophages of the M2 type significantly increase in IUA, promoting endometrial stromal cell-myofibroblast transdifferentiation through the GAS6/AXL/NF-κB signaling pathway. In contrast, removing CD301 macrophages can reverse endometrial fibrosis *in vivo* and *in vitro*, improving pregnancy outcomes (Lv H. et al., 2023). Research indicates that STAT1-dependent cytokine production from M1 macrophages propagates inflammation, accelerating both the inflammatory cascade and endometrial adhesion pathogenesis (Peng et al., 2025). Most studies have indicated that macrophages are key factors in promoting endometrial fibrosis (Sun et al., 2024; Liu D. et al., 2024; Wang et al., 2021; Wang et al., 2024). Existing research has established that circRNAs contribute to fibrotic progression of various diseases by regulating macrophage activation, polarization, and inflammatory response (Zhou et al., 2018) (Hou et al., 2023) (Fu et al., 2022; Lin et al., 2023). It implies that circRNAs may also form intrauterine adhesions by regulating macrophages. Through bioinformatics research, we found five circRNAs significantly associated with the proportion of M1 macrophages, which are hsa-PLOD2_0001, hsa-FAM13B_0009, hsa-ARHGAP26_0021, hsa-GPBP1_0024, and hsa-UBQLN1_0018. Among these, hsa-PLOD2_0001 and hsa-FAM13B_0009 show the most significant correlation. The circRNA hsa-PLOD2_0001 is generated through exon 2–7 circularization of the PLOD2 pre-mRNA, which encodes procollagen-lysine, two-oxoglutarate 5-dioxygenase 2. CircPLOD2 contributes to pericyte-mediated fibrotic responses under hypoxic conditions through enhanced ECM collagen deposition, as demonstrated by recent findings (Glaser et al., 2023). Studies of osteosarcoma also reveal that PLOD2 upregulation is linked to enhanced macrophage recruitment within the tumor microenvironment (Trang et al., 2023). These indicate that the tissue fibrosis caused by circPLOD2 may be related to macrophages. Hsa-FAM13B_0009 is formed by the circularisation of exons 8 to 10 of FAM13B, with a splice length of 331 bp. Elevated circFAM13B expression levels were observed in endometrial tissues from women experiencing recurrent implantation failure (Zhang et al., 2023). Research shows that the dysregulation of circFAM13B has potential value as a biomarker for cancer treatment, such as its ability to suppress the proliferation of bladder cancer by altering the tumor immune microenvironment through the inhibition of glycolysis (Lv J. et al., 2023). Then, the polarization of M1 macrophages in the tumor immune microenvironment is a key factor hindering cancer development (Deng et al., 2023), suggesting that circFAM13B may promote M1 macrophage polarization. Therefore, circFAM13B may induce inflammatory responses and IUA formation by promoting M1 macrophage polarization. We used RT-qPCR detection to verify the high expression of hsa-PLOD2_0001 and hsa-FAM13B_0009 in the endometrium of IUA patients, which may be closely related to endometrial fibrosis.

No research has focused on the abnormal regulation of circRNA splice site usage during IUA. To our knowledge, we have conducted the first analysis of the detailed map of circRNA linear splice sites and selectively regulated back-splicing. Analysis has shown that the genes associated with CAS are predominantly enriched in biological pathways related to protein localization to cilia, the EGFR signaling pathway, the regulation of epithelial to mesenchymal transition, chorion development, the progesterone receptor signaling pathway, targeted protein delivery, and the ERBB signaling pathway. The EGFR signaling pathway is essential for modulating cellular processes such as proliferation, migration, and viability, contributing to normal tissue regeneration. However, excessive EGFR signaling in IUA patients may promote uncontrolled tissue proliferation and subsequent fibrosis development. Additionally, following uterine damage, such as surgery or infection, the EMT pathway may be induced as part of tissue repair. However, inappropriate or excessive EMT may lead to an overabundance of interstitial cells and fibrosis, promoting the formation of IUA. We identified three genes in the EGFR signaling pathway and the EMT pathway, namely, GAB1, PTK2, and APLF. GAB1 is an adaptor protein that becomes tyrosine-phosphorylated upon activation by diverse extracellular signals, including growth factors, cytokines, G protein-coupled receptor agonists, and various immune antigens. This modification enables its interaction with Grb2, SHP-2, and the p85 regulatory subunit of PI3K, thereby triggering distinct downstream signaling cascades. GAB1 is overexpressed in multiple cancers and fibrotic tissues, participating in cell adhesion, metastasis, and activation (Chen et al., 2025). Research shows that E-cadherin can mediate the adhesion of epithelial cells through Src family kinases, inducing Gab-1 phosphorylation (Shinohara et al., 2001). PTK2 integrates multiple signaling inputs, from ECM adhesion to growth factor stimulation, to coordinate cell behavior. This non-receptor tyrosine kinase exerts dual functionality through enzymatic activity and protein scaffolding to regulate adhesion, motility, and viability (Le Coq et al., 2022). PTK2 participates in the progression of different tumors and fibrotic diseases through multiple mechanisms, such as regulating cell adhesion, migration, survival, EMT, and angiogenesis. Research shows that PTK2 can also mediate the continuous progression of endometriosis by promoting inflammatory responses and tissue fibrosis (Nagai et al., 2020). APLF serves as a scaffolding protein that enhances non-homologous end joining-mediated repair of DNA double-strand breaks, while also supporting base/nucleotide excision repair of single-strand lesions. Most studies indicate that APLF aids tumor cells in evading the lethal effects of radiotherapy and chemotherapy (Wu et al., 2024). Research has shown that the downregulation of APLF in mouse embryonic fibroblasts promotes reprogramming and improves induced pluripotent stem cell (iPSC) induction efficiency through E-cadherin upregulation (Syed et al., 2016). Since loss of E-cadherin impairs intercellular adhesion and induces EMT, we hypothesize that APLF may promote this transition by downregulating E-cadherin. Moreover, through bioinformatics research, we took the intersection of the circRNAs exhibiting differential expression in IUA compared to normal samples and those that underwent differential alternative splicing, resulting in 10 circRNAs: hsa-SFMBT2_0011, hsa-PGR_0008, hsa-ZZZ3_0002, hsa-MTCL1_0019, hsa-KLHL24_0011, hsa-GAB1_0008, hsa-DCUN1D4_0001, hsa-SEC31A_0019, hsa-GPBP1_0024, and hsa-KMT2C_0040. The expression level of KLHL24 has been confirmed in IUA tissues.

There is no literature to study the regulatory role of RBPs in IUA. However, emerging evidence indicates that RBPs modulate key endometrial cellular processes, including proliferation, differentiation, and inflammatory activation. Furthermore, these proteins influence ECM dynamics through degradation and reorganization, as exemplified by CSDC2, CIRP, PTBP1, YB-1, HuR, and AUF1 (Vallejo et al., 2018; Hamid et al., 2003; Zhao et al., 2022; Rybalkina and Moiseeva, 2022; Khalaj et al., 2017; Ing, 2010). Additionally, the formation and progression of IUA involves multiple biological processes, including endometrial cell proliferation/differentiation, inflammatory activation, and ECM remodeling (Ang et al., 2023). These suggest that RBPs may play an essential role in developing IUA. We identified a total of 16 DERBPs between the two groups. Additional co-expression analysis of DERBPs and CAS events identified EXO1, SORBS1, ENOX1, P2RX7, KPNA2, TPX2, and DDX43 as potential upstream RBPs for two EGFR signaling pathway-associated CAS events: clualt5p1141:GAB1 and clualt5p1561:PTK2. The expression levels of EXO1 and SORBS1 were confirmed by RT-qPCR and Western blot analysis. EXO1 is a multifunctional protein possessing both 5′-3′ exonuclease and ribonuclease activities, playing essential roles in crucial DNA metabolic pathways including mismatch repair (MMR), homologous recombination (HR), double-strand break repair (DSBR), and genomic replication. Dysregulation of EXO1 has been implicated in various pathological conditions, particularly cancer. Previous investigations (Zhang Y. L. et al., 2024; Trubicka et al., 2017) demonstrated that EXO1 and GAB1 cooperate synergistically in modulating tumor development and oxidative stress. These findings suggest that EXO1’s DNA repair capacity could influence the genomic stability of GAB1-associated signaling pathways. The SORBS1 gene encodes a protein featuring both Sorbin and SH3 domains. This protein participates in the regulation of cell adhesion, cytoskeletal formation, the insulin signaling pathway, and tumor metastasis. Research demonstrates that SORBS1 suppresses EMT in breast cancer cells, concurrently controlling malignant behaviors including cell proliferation, motility, and invasiveness through PI3K/AKT pathway regulation (Feng et al., 2024).

This study also has limitations: first, the sample size is relatively small; circRNA profiling and associative analyses were based on seven pairs of IUA and normal endometrial samples from the GEO database, and only six pairs of clinical samples were used for subsequent validation, which may limit the generalizability of the observed associations. Second, the current study focuses on exploring expression associations and has not elucidated causal regulatory mechanisms,for example, the causal relationship between circRNA hsa-KDM4B_0007 and hsa-miR-665 has not been clearly established; further research is needed to investigate the role of these associations in IUA pathogenesis. Third, the specific molecular mechanisms underlying the association between circRNAs and macrophages remain unclear. Additionally, ceRNA/RBP regulatory relationships were inferred using computational algorithms, which require experimental validation to confirm biological relevance. In the future, expanding the sample size and conducting mechanistic studies will help improve the reliability and translational value of the study conclusions.

## Conclusion

To our knowledge, this study is the first to conduct genome-wide profiling and functional annotation of circRNAs associated with IUA, systematically revealing their multidimensional regulatory roles in the pathogenesis of IUA. The identified associative patterns—including those between circRNAs and uterine contraction/cell cycle, between circRNAs and M1 macrophage polarization, and between circRNA splicing events and RBPs—offer potential candidates for the screening of IUA biomarkers and the exploration of therapeutic targets.

## Data Availability

The original contributions presented in the study are included in the article/Supplementary Material, further inquiries can be directed to the corresponding authors. Processed data, intermediate files, and customized analysis code have been deposited in Figshare and are publicly available at https://doi.org/10.6084/m9.figshare.31442254.
