# Supplementary material for: Integrative circRNA landscape of intrauterine adhesions: putative ceRNA axes and circRNA-associated splicing usage linked to contractility and immunity
Source: Front Mol Biosci. 2026 May 7;13:1763980. doi: 10.3389/fmolb.2026.1763980 (PMC13189722; doi:10.3389/fmolb.2026.1763980)
Supplement: Supplementary file 1 [file Supplementaryfile1.pdf]

Add supplementary images and descriptions

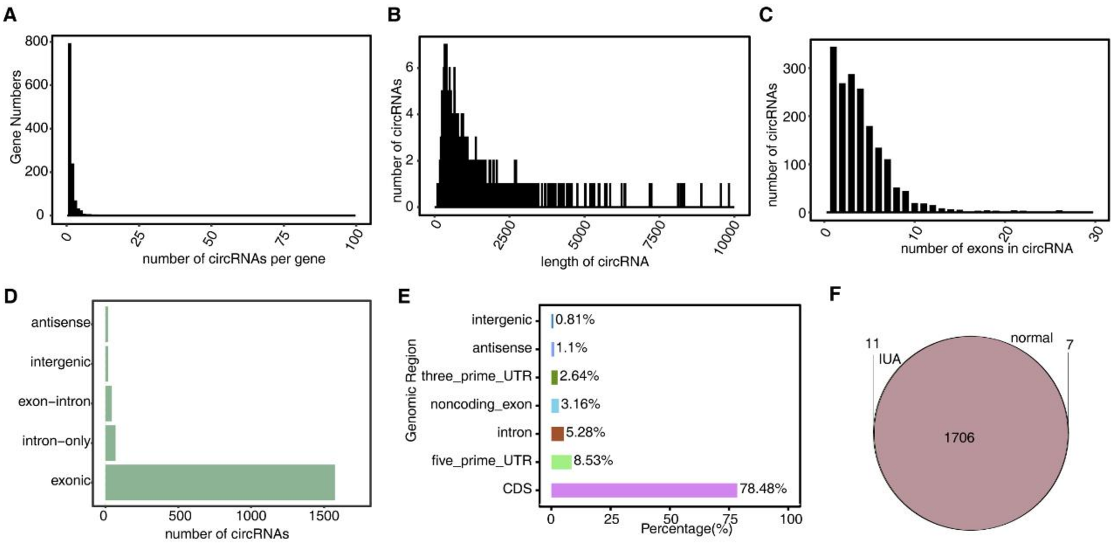

Figure S1. CircRNA Expression Dynamics in IUA Pathogenesis.

A. Histogram showing the distribution of genes with different number of detected circRNAs.

B. Histogram showing the distribution of circRNAs with different length.

C. Based on the recognition results by FcircSEC, the bar plot showing the number of exons contained in circRNAs.

D. The bar graph showing the number of candidates circRNA with different type.

E. The bar graph showing the number of candidates circRNA located on different genomic regions.

F. The Venn diagram illustrates the intersection of circRNAs detected in the IUA or normal groups.

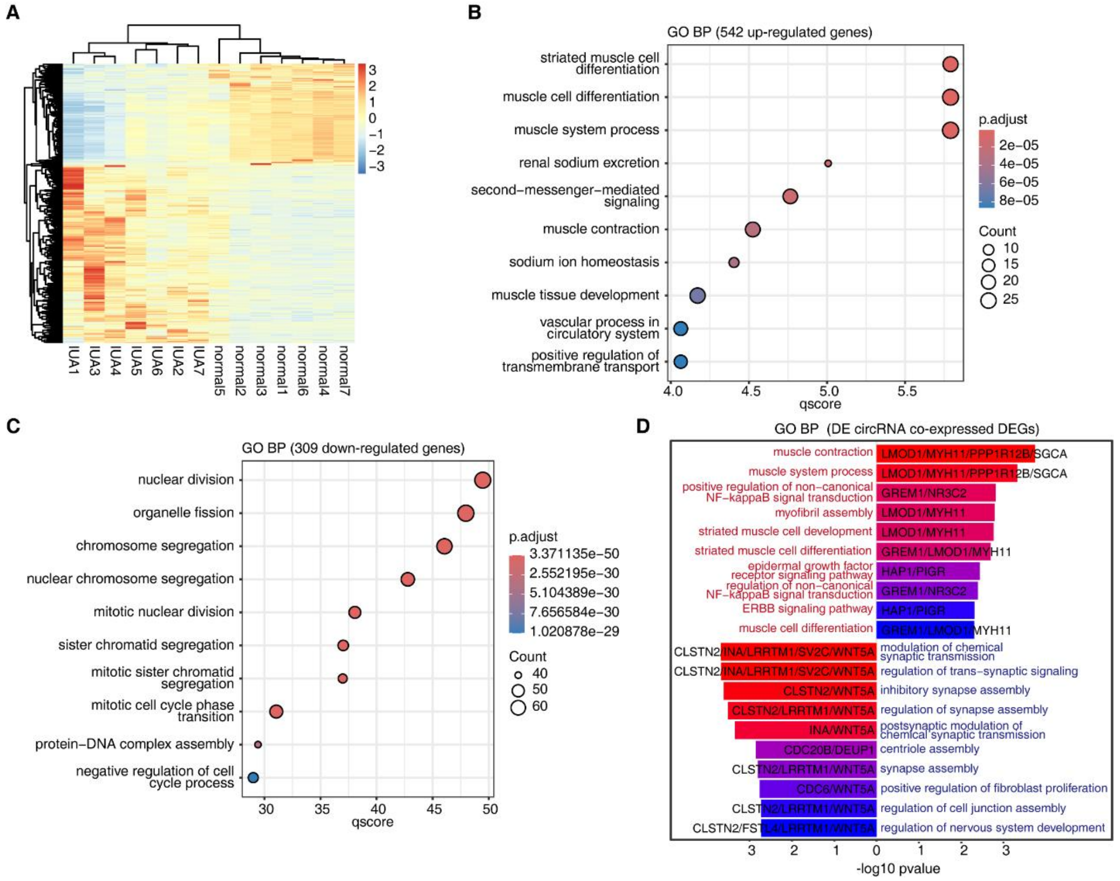

Figure S2. Construction and Functional Implication of circRNA-miRNA-mRNA Network in IUA.

A. The heatmap diagram showing the expression profile of DEGs.

B. Dot plot showing the most enriched GO biological process results of host genes of up regulated DEGs in IUA group.

C. Dot plot showing the most enriched GO biological process results of host genes of down regulated DEGs in IUA group.

D. Bar plot showing the most enriched KEGG pathways of up-regulated and down-regulated DEGs of circRNA-miRNA-mRNA network in figure A-B.

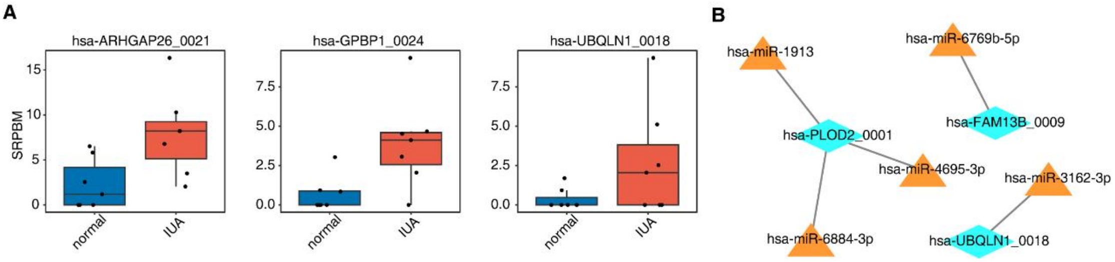

Figure S3. Immune Cell Infiltration and Its Correlation with Differentially Expressed circRNAs in IUA.

(A). Boxplot showing expression level (SRPBM) of circRNA hsa-ARHGAP26_0021, has-GPBP1_0024 and hsa-UBQLN1_0018.

(B). MiRNA-circRNA network. Predictions of miRNA-circRNA target relationships were made using both Miranda and Rnahybrid.

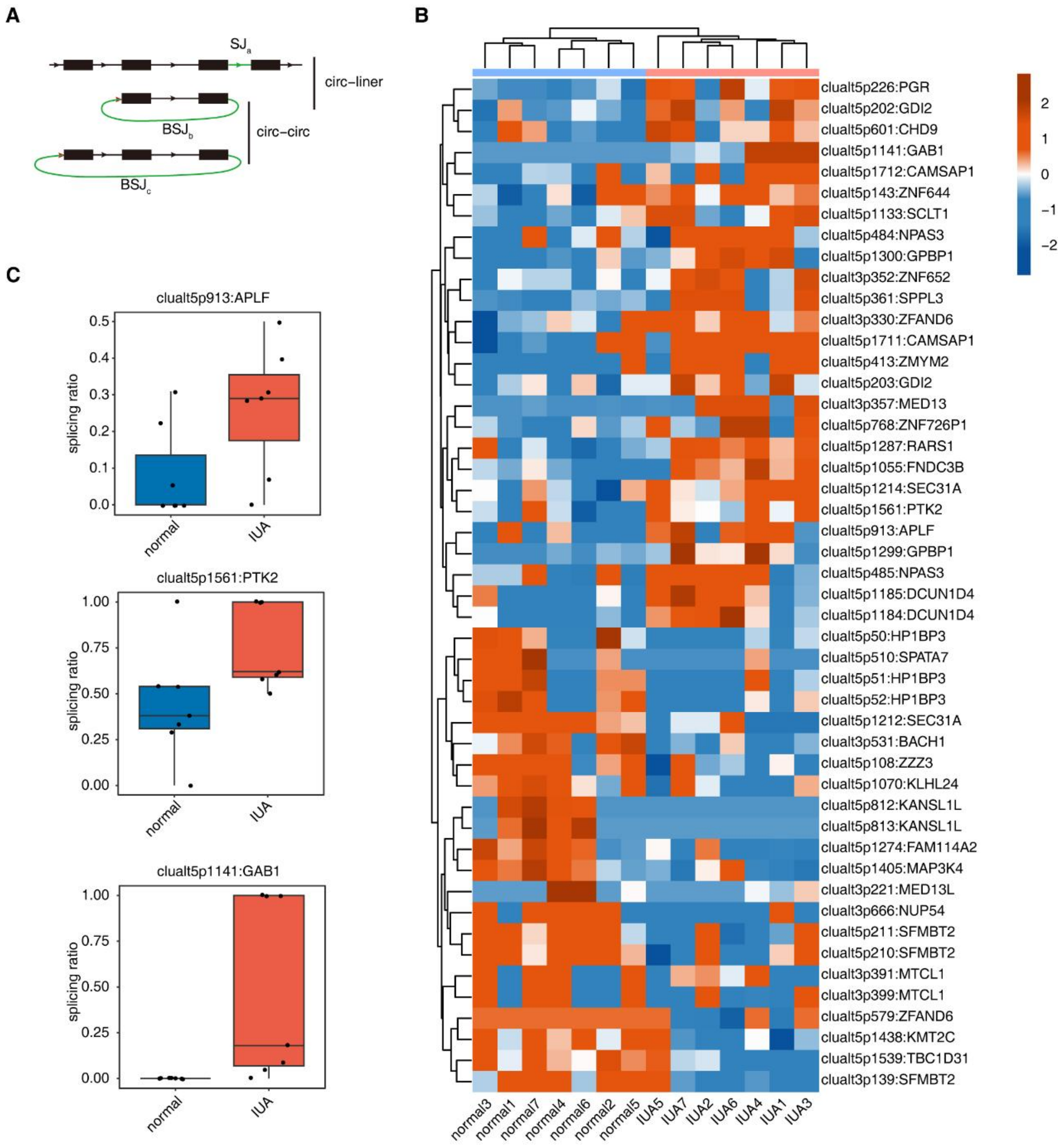

Figure S4. Identification of highly conserved alternative splice sites usage of circular RNA in IUA.

A. We categorized CAS events into two distinct models based on splicing site usage variation, as defined by SUVA. Each model consists of two paired splice junctions (SJ). The "circ-liner" model signifies one alternative splice site is linearly spliced, while the other splice site is back-spliced. The "circ-circ" model indicates that both alternative splice sites are back-spliced.

B. The heatmap diagram offers a graphical representation of the splicing ratio profile of CAS events.

C. The boxplot visually represents the splicing ratio of three CAS events across all samples.
