# Supplementary figures and images for: Integrative circRNA landscape of intrauterine adhesions: putative ceRNA axes and circRNA-associated splicing usage linked to contractility and immunity
Source: Front Mol Biosci. 2026 May 7;13:1763980. doi: 10.3389/fmolb.2026.1763980 (PMC13189722; doi:10.3389/fmolb.2026.1763980)

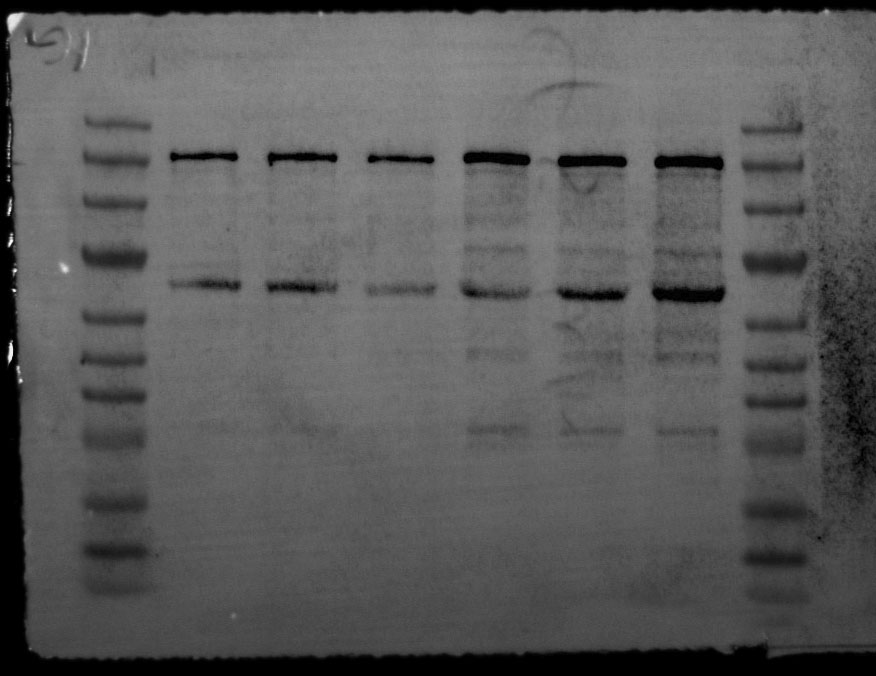

Supplement: Supplementary file 3 [file Supplementaryfile3.zip › IUA-WB Original image/CD163原图.jpg]

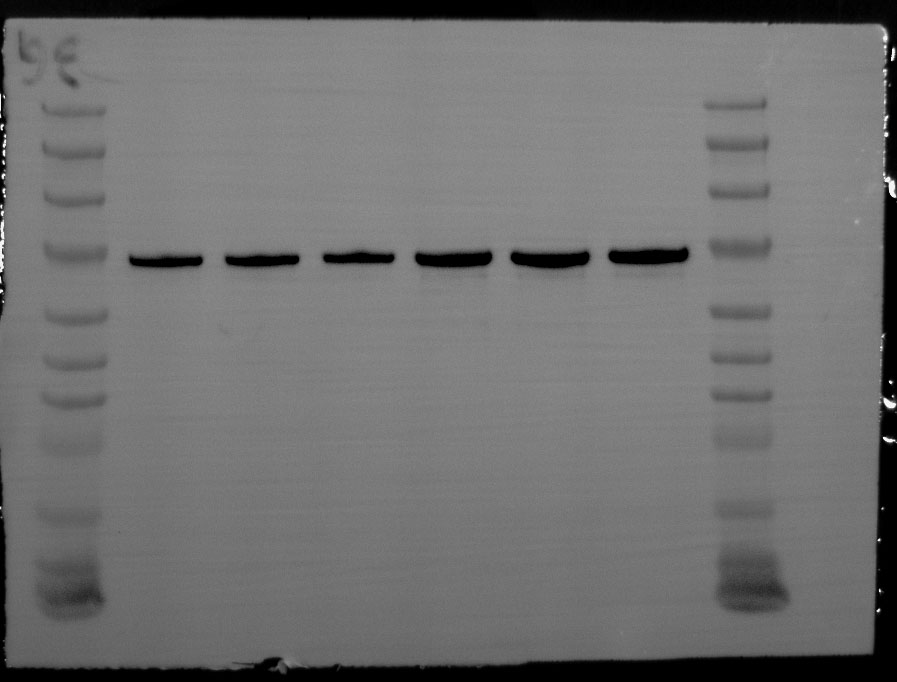

Supplement: Supplementary file 3 [file Supplementaryfile3.zip › IUA-WB Original image/CD68原图.jpg]

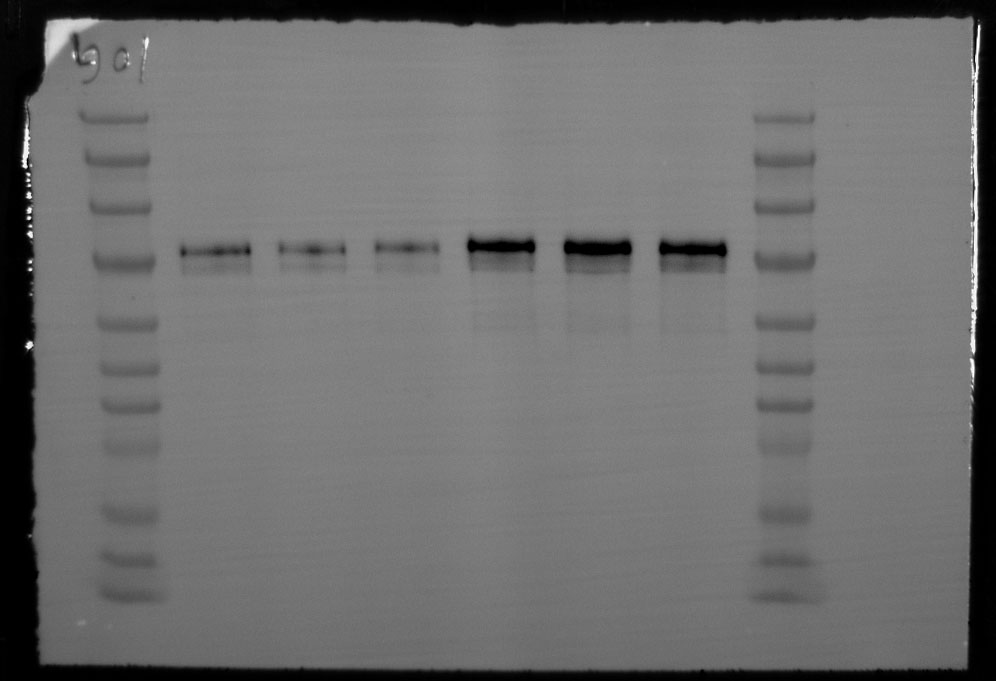

Supplement: Supplementary file 3 [file Supplementaryfile3.zip › IUA-WB Original image/CD86原图.jpg]

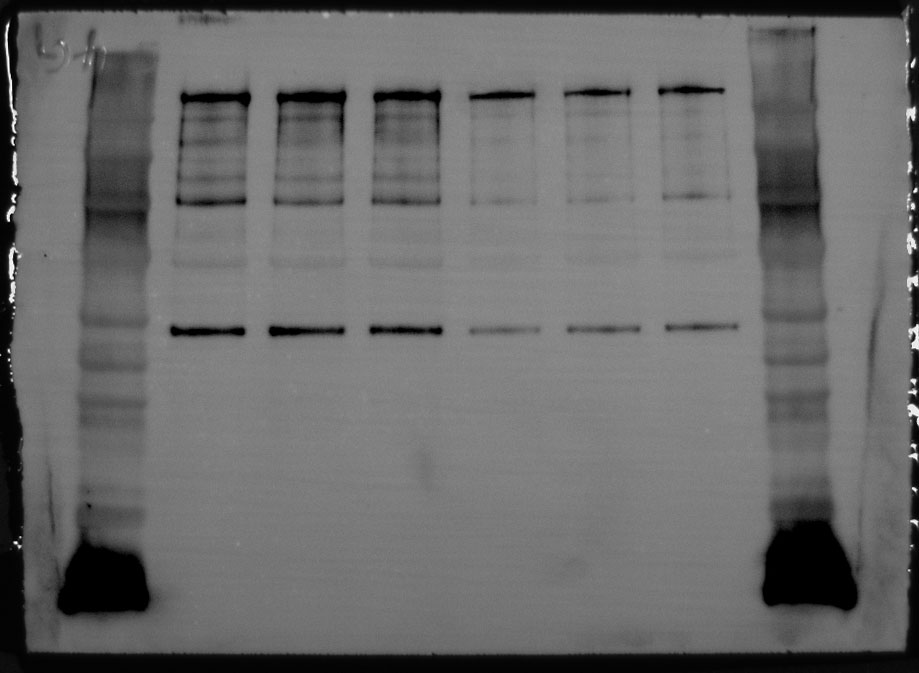

Supplement: Supplementary file 3 [file Supplementaryfile3.zip › IUA-WB Original image/Ki67原图.jpg]

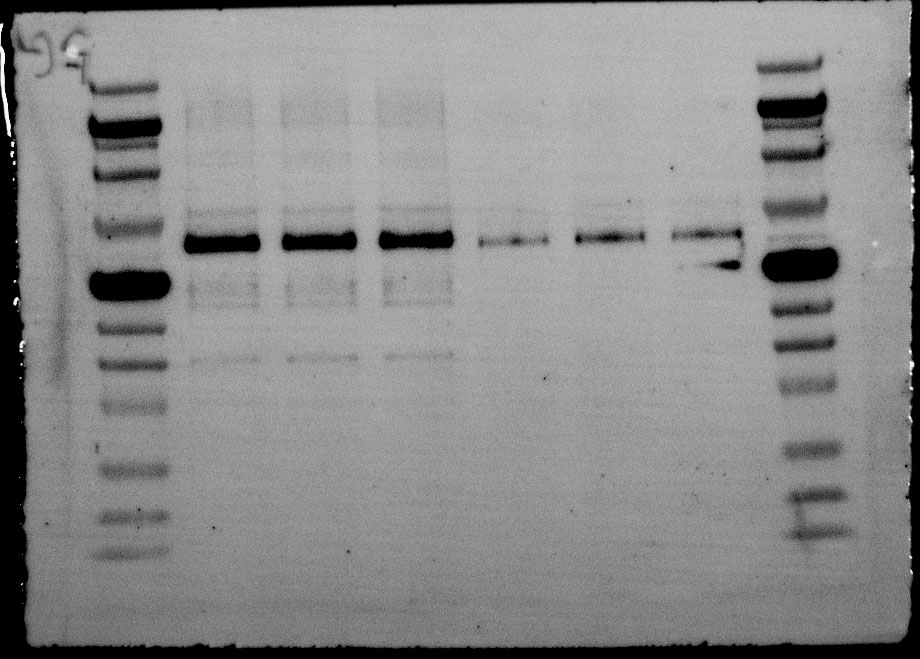

Supplement: Supplementary file 3 [file Supplementaryfile3.zip › IUA-WB Original image/KLHL24原图.jpg]

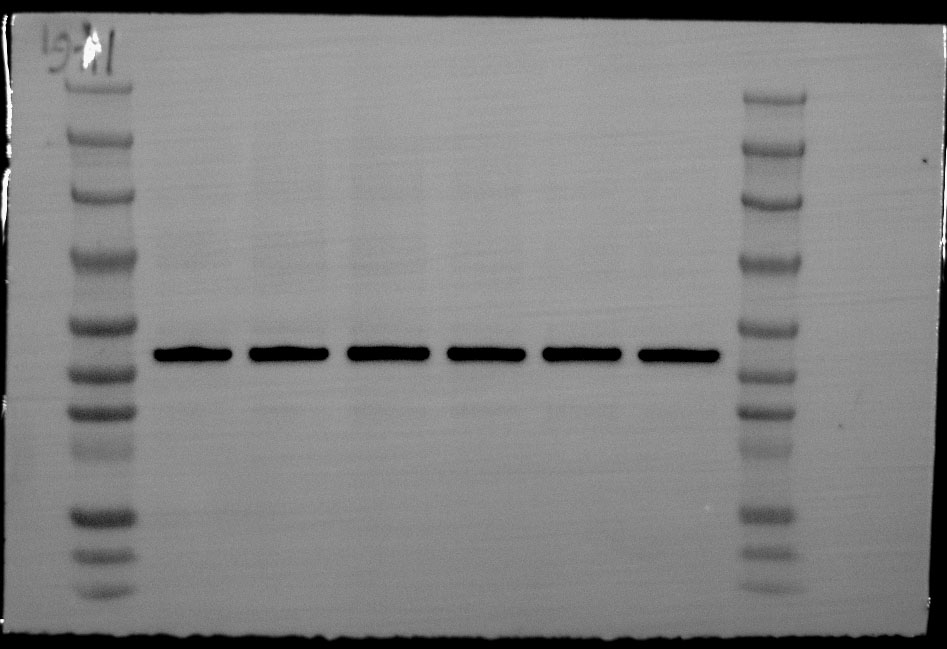

Supplement: Supplementary file 3 [file Supplementaryfile3.zip › IUA-WB Original image/β-actin1原图.jpg]

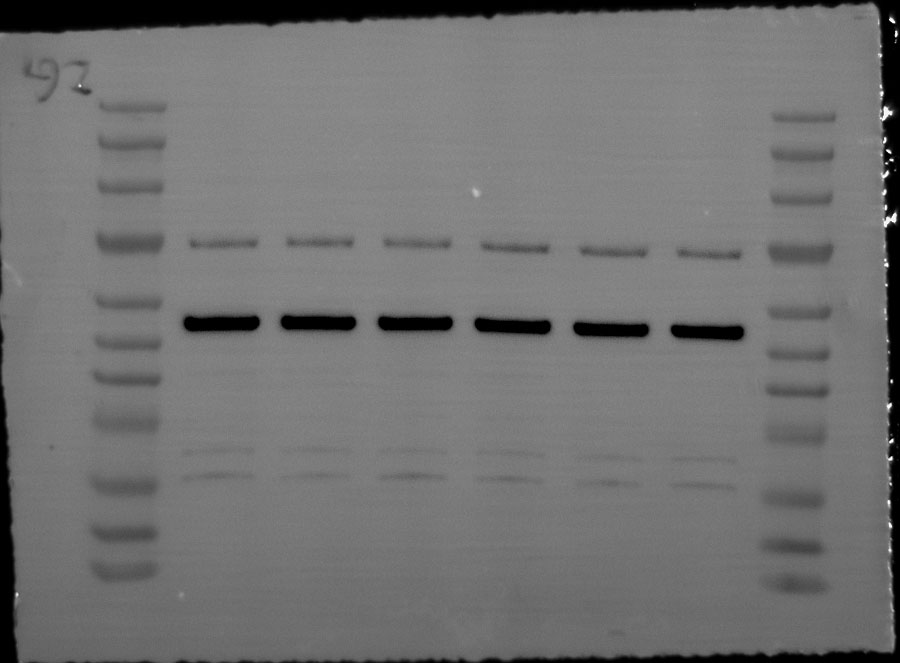

Supplement: Supplementary file 3 [file Supplementaryfile3.zip › IUA-WB Original image/β-actin2原图.jpg]
