# Supplementary material for: Integrative circRNA landscape of intrauterine adhesions: putative ceRNA axes and circRNA-associated splicing usage linked to contractility and immunity
Source: Front Mol Biosci. 2026 May 7;13:1763980. doi: 10.3389/fmolb.2026.1763980 (PMC13189722; doi:10.3389/fmolb.2026.1763980)
Supplement: Supplementary file 4 [file Supplementaryfile4.pdf]

Figure 1. Original western blot for three repeats

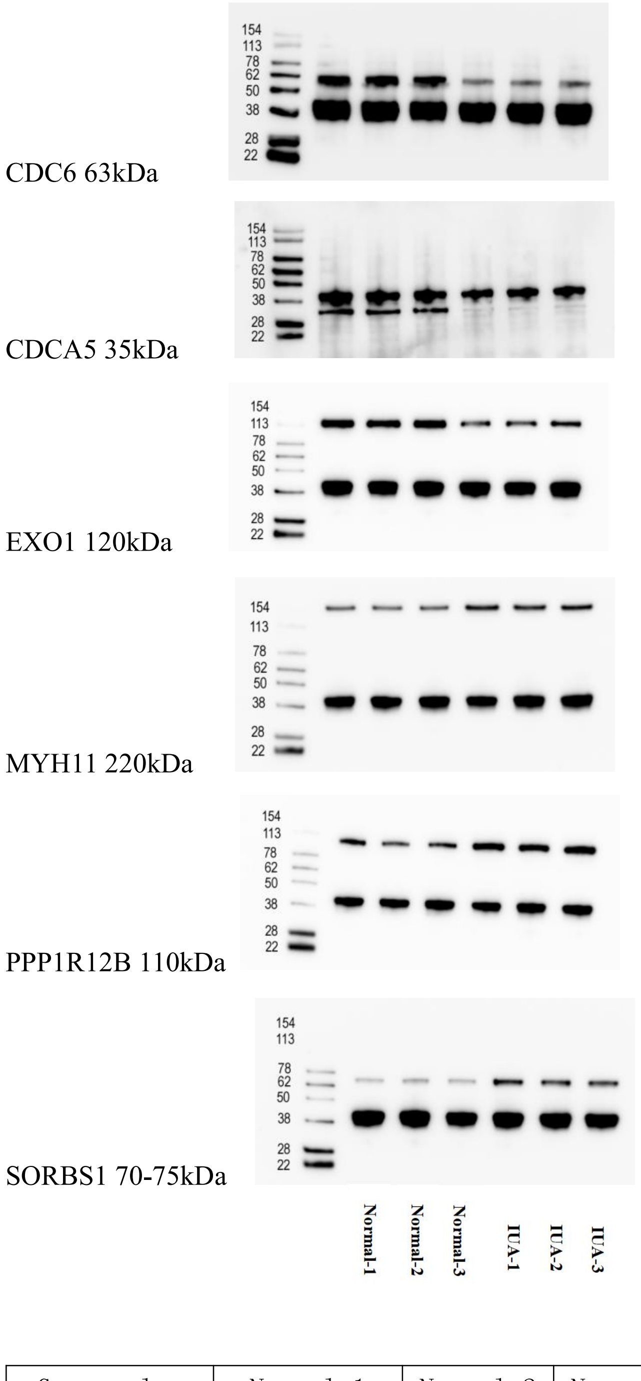

Gray value	Normal-1	Normal-2	Normal-3	IUA-1	IUA-2	IUA-3
MYH11	525	509	567	1021	915	932
β-actin	1927	2031	2025	2031	2090	2105
PPP1R12B	858	735	794	1496	1485	1542
β-actin	1628	1663	1707	1741	1728	1787
CDC6	2049	2081	2024	959	1013	1085
β-actin	3648	3562	3493	3483	3450	3423
CDCA5	1229	1155	1190	220	305	206

β-actin	2586	2573	2561	2415	2434	2468
EXO1	1568	1492	1652	775	779	864
β-actin	2481	2506	2607	2552	2436	2571
SORBS1	235	305	290	1014	965	995
β-actin	2657	2684	2527	2624	2634	2601
